# Recent Advances and Applications of Chitin and Chitosan Hydrogel Scaffolds in Tissue Engineering

**DOI:** 10.3390/gels12050427

**Published:** 2026-05-13

**Authors:** A. M. Abdel-Mohsen, Rasha M. Abdel-Rahman, Katerina Skotnicova

**Affiliations:** 1 Faculty of Materials Sciences and Technology, VSB—Technical University of Ostrava, 70800 Ostrava, Czech Republic; 2 Institute of Materials Chemistry, Faculty of Chemistry, Brno University of Technology, Purkyova 464/118, 61200 Brno, Czech Republic; 3 Central European Institute of Technology, Brno University of Technology, Purkyova 656/123, 61200 Brno, Czech Republic

**Keywords:** hydrogel, chitosan, extraction, characterization, applications, 3D scaffold, tissue regeneration

## Abstract

Hydrogel scaffolds have emerged as a central platform in tissue engineering due to their ability to mimic the extracellular matrix and support cellular functions. Among natural polymers, chitin and its derivative chitosan have emerged as valuable candidates for hydrogel scaffold development because of their biodegradability, compatibility with living tissues, and inherent biological functionality; however, their distinct and complementary roles in hydrogel scaffold design are often insufficiently differentiated in the literature. This review provides a comprehensive and mechanism-driven analysis of chitin- and chitosan-based hydrogel scaffolds, emphasising how their molecular structure governs network formation, mechanical performance, and biological functionality. Chitin is highlighted primarily as a structurally robust and crystalline component suitable for reinforcement. In contrast, chitosan serves as a versatile, soluble, and chemically reactive matrix enabling various crosslinking and functionalization strategies. Recent advances in physical, ionic, and covalent crosslinking as well as composite scaffold engineering, biofunctionalization, and emerging fabrication approaches such as injectable systems and three-dimensional bioprinting are systematically examined. The relationships between scaffold architecture, degradation behaviour, and cellular responses are discussed in key tissue engineering applications, including bone, cartilage, skin, and nerve regeneration. Importantly, this review introduces a unified structure–property–function framework that distinguishes the roles of chitin and chitosan within hydrogel systems and links crosslinking mechanisms to application-specific performance requirements, an aspect not comprehensively addressed in previous studies. Current challenges related to mechanical limitations, material variability, and clinical translation are critically evaluated, and future perspectives for the rational design of next-generation biomimetic hydrogel scaffolds are proposed.

## 1. Introduction

Tissue engineering integrates biology, materials science, and engineering to restore, maintain, or replace damaged tissues and organs. Central to this field is the development of functional tissue substitutes that replicate not only structural architecture but also biological activity [1,2]. Successful regeneration depends on engineered constructs capable of supporting cell survival, proliferation, and differentiation while facilitating dynamic interactions with host tissues [3,4,5]. As a result, scaffold materials must have finely tuned mechanical properties, interconnected porosity for nutrient and waste transport, controlled biodegradation, and the ability to present biochemical and biophysical signals essential for tissue development [6]. Among scaffold systems, hydrogels are particularly attractive because their hydrated three-dimensional networks closely resemble the native extracellular matrix. Their physicochemical characteristics can be adjusted to control stiffness, swelling behaviour, and degradation kinetics while also enabling cell encapsulation and localised delivery of signalling molecules. However, the functional performance of hydrogels is strongly influenced by the selected polymer system and network fabrication strategy, making material design a critical determinant of scaffold efficacy [7,8].

Natural polysaccharides have gained significant attention due to their biocompatibility and intrinsic biological relevance [9,10,11]. Within this group, chitin and chitosan represent structurally related but functionally distinct biomaterials. Chitin, the second most abundant natural polysaccharide after cellulose, is derived primarily from the exoskeletons of crustaceans and insects as well as fungal cell walls [12,13,14].

Chitin exhibits a highly crystalline and ordered structure that confers mechanical strength, although it is limited by its poor processability. Chitosan, in contrast, is more soluble and chemically versatile because of its reactive amino groups, allowing extensive crosslinking and functional modification. Together, these complementary characteristics enable the design of hydrogel scaffolds that integrate structural reinforcement with biochemical adaptability [15,16]. Structurally analogous to glycosaminoglycans present in the ECM, chitosan shows low immunogenicity, intrinsic antimicrobial activity, hemostatic properties, and the capacity to promote wound healing. These characteristics make chitin and chitosan particularly attractive candidates for the fabrication of hydrogel-based scaffolds [17,18]. Recent advances in crosslinking strategies, composite scaffold engineering, nanostructural modification, and biofunctionalization have significantly improved the mechanical performance, structural stability, and regenerative efficacy of chitin and chitosan-based hydrogels [19,20,21]. Furthermore, emerging fabrication technologies such as injectable in situ gelation systems and three-dimensional bioprinting have expanded their translational potential [22,23].

This review provides a comprehensive analysis of recent developments in the design, fabrication, and functional optimisation of chitin- and chitosan-based hydrogel scaffolds. Emphasis is placed on their physicochemical properties, structural engineering strategies, and applications in bone, cartilage, skin, nerve, and other tissue regeneration contexts. In addition, it clarifies the distinct but complementary roles of chitin and chitosan in hydrogel systems and relates crosslinking methods to the specific functional requirements of different tissue engineering applications, an area that has received limited systematic attention in previous research.

## 2. Chemistry and Fundamental Properties of Chitin and Chitosan

### 2.1. Molecular Structure

Chitin is a linear natural biomaterial consisting of β-(1 → 4)-linked N-acetyl-D-glucosamine (GlcNAc) units [13,24,25,26]. Structurally analogous to cellulose, chitin differs by the presence of an acetamide group at position C-2, which contributes to strong intermolecular hydrogen bonding and high crystallinity [27,28,29,30]. This structural organisation results in limited solubility in common solvents and significant mechanical rigidity [31,32,33,34]. Chitin present in three polymorphic forms: α-chitin, β-chitin, and γ-chitin, distinguished by the arrangement of polymer chains. α-Chitin, the most abundant and stable form, exhibits antiparallel chain wrapping and is found mainly in crustacean shells. *β*-Chitin, with parallel chain alignment, is present in squid pens and shows greater reactivity and swelling capacity. γ-Chitin contains a mixture of parallel and antiparallel orientations [33,35,36,37]. Chitosan is derived from chitin through partial alkaline deacetylation, converting N-acetyl-D-glucosamine units into D-glucosamine residues. The resulting copolymer consists of randomly distributed glucosamine and acetylated units [34,37,38,39,40,41]. The degree of deacetylation (DDA, which typically varies from 50% to 95%) is a critical parameter that influences solubility, charge density, degradation rate, and biological performance [37,42,43,44,45,46,47]. A higher DDA increases the availability of free amino groups, improves solubility in acidic medium, and facilitates chemical modification [38]. The molecular weight (MW) of chitosan, which can range from a few micrometres to over 1000 micrometres depending on the processing conditions, also significantly affects viscosity, mechanical strength, and degradation behaviour. Therefore, both DDA and MW must be carefully controlled for biomedical applications [48,49].

### 2.2. Influences of Sources and Extraction Processes on the Performance of Chitin and Chitosan Hydrogels

The biological and biomedical performance of chitin and chitosan hydrogels is significantly influenced by the origin of the raw material (e.g., crustacean shells, squid pens, fungal cell walls, or insect exoskeletons) and the conditions used during extraction and derivatization. These factors affect critical properties such as molecular weight (MW), degree of deacetylation, crystallinity, purity, and residual protein content, which in turn determine biocompatibility, mechanical strength, degradation kinetics, antimicrobial activity, immunogenicity, and cell–material interactions [50,51]. Crustacean-derived chitin generally has a higher molecular weight and crystallinity than fungal or insect sources. However, conventional harsh extraction methods involving high concentrations of NaOH and HCl at elevated temperatures often cause partial depolymerisation, uncontrolled deacetylation, and the introduction of impurities. This results in batch-to-batch variability that directly compromises hydrogel performance: lower MW chitosan typically produces mechanically weaker gels with faster degradation rates, while higher DD increases cationic charge density, enhancing antimicrobial efficacy and electrostatic interactions with cells and extracellular matrix components [52,53]. Milder, more sustainable extraction approaches, such as enzymatic deproteinization, ionic liquids, or deep eutectic solvents (DESs), better preserve the native polymer chain length and reduce contaminants. These methods produce chitosan with higher MW, more consistent DDA, and lower immunogenicity, leading to hydrogels with improved mechanical integrity, predictable degradation profiles, and enhanced biocompatibility [54,55]. For example, DES-based extraction has been shown to produce high-purity chitin while minimising environmental impact and polymer degradation [56]. Chemical derivatization strategies further modulate biomedical behaviour. Quaternization improves the solubility of water and antibacterial activity, PEGylation enhances hemocompatibility and reduces nonspecific protein adsorption, and grafting with other polymers or bioactive molecules tailors the degradation rate and cell adhesion [50,57]. Source-dependent differences in chitin allomorphs (α-, β-, γ-chitin) also influence the resulting hydrogel microstructure, porosity, swelling behaviour, and ultimately nutrient diffusion, cell infiltration, and tissue integration [11,29,30,37,51].

### 2.3. Physicochemical Properties

Chitin is largely insoluble in water and most organic solvents due to its extensive hydrogen bonding and high crystalline structure. Conversely, chitosan is soluble in dilute acidic solutions (pH < 6.5), where protonation of the primary amino groups (-NH_2_ -NH_3_^+^) makes it a cationic polyelectrolyte [33]. This positive charge enables electrostatic interactions with negatively charged biomolecules, glycosaminoglycans, and cell membranes [58,59]. Chitin exhibits high crystallinity and mechanical strength, while chitosan generally exhibits lower crystallinity due to structural disruption [13]. Although chitosan hydrogels provide flexibility and a high-water content, their intrinsic mechanical strength is limited, necessitating crosslinking or composite reinforcement for load-bearing applications [20,60]. Chitosan-based hydrogels exhibit a substantial swelling capacity due to their hydrophilic hydroxyl and amino groups [21]. The swelling behaviour depends on the crosslink density, pH, ionic strength, and DDA. This property is critical for nutrient diffusion, drug loading, and cell infiltration in tissue engineering scaffolds [16,61]. Chitosan is biodegradable through enzymatic hydrolysis, mediated primarily by lysozyme and other glycosidases that cleave β-(1 → 4) linkages [62,63]. The degradation rate is influenced by DDA, MW, crystallinity, and crosslinking density [63]. Importantly, the degradation product, mainly oligosaccharides, is non-toxic and can be metabolised or excreted [39,64].

### 2.4. Biological Properties

Chitin and chitosan exhibit excellent biocompatibility with minimal cytotoxicity and low immunogenicity [65]. Their structural similarity to glycosaminoglycans supports favourable cell-material interactions, promoting adhesion and proliferation in various cell types [66]. Chitosan has intrinsic antimicrobial properties attributed to its cationic nature [67]. Electrostatic interactions between protonated amino groups and negatively charged microbial membranes alter membrane integrity, leading to leakage of intracellular components [68]. This characteristic is particularly advantageous for wound healing and infection-prone implant sites [69]. In addition, chitosan promotes platelet adhesion and aggregation, facilitating rapid clot formation. It also stimulates macrophage activation and cytokine production, thus accelerating wound healing and tissue repair [15,70]. Recent studies indicate that chitosan can modulate immune responses depending on the MW and DDA, influencing macrophage polarisation and inflammatory signaling pathways [64]. This property offers potential for controlled immunoregulation in regenerative applications [20,38]. A key advantage of chitosan is the presence of reactive functional groups, including primary -NH_2_ groups at the C-2 position and primary and secondary -OH groups at the C-3 and C-6 positions [13]. These functional groups provide versatile sites for chemical modification, allowing the grafting of bioactive peptides, the introduction of crosslinkable moieties and conjugation with therapeutic agents or growth factors [38,71]. Furthermore, they enable the formation of chitosan derivatives such as carboxymethyl chitosan [72], quaternized chitosan [73], and methacrylate chitosan [72]. Such chemical modifications not only improve solubility and mechanical properties but also impart stimuli-responsive behaviour, making chitosan highly adaptable for the design of advanced hydrogel scaffolds in tissue regeneration applications [74,75].

### 2.5. Mechanism- and Property-Based Functional Classification of Chitin and Chitosan Hydrogels

Chitin and chitosan-based hydrogels can be classified according to their network formation mechanisms, which directly influence their structural, mechanical, and functional properties [21]. Physically crosslinked hydrogels rely on noncovalent reversible interactions such as hydrogen bonding [76], hydrophobic interactions, or ionic associations [21], making them suitable for applications that require mild processing [75], injectability [76], or stimulus responsiveness [21]. However, their mechanical weakness and sensitivity to environmental conditions often limit long-term stability [76,77]. Chitin nanowhisker hydrogels represent a distinct class in which network formation is governed by the percolation of rigid nanocrystals [78,79] (Table 1). These hydrogels achieve high stiffness and optical transparency at low solid contents, serving effectively as reinforcing frameworks or functional fillers rather than as standalone soft materials [80,81].

Chitosan-based physical and ionic hydrogels exploit the presence of protonatable amine groups, allowing pH-triggered gelation and reversible ionic crosslinking [82,83]. This chemical versatility allows precise control over swelling behaviour [84] and mechanical performance [85], which is particularly advantageous for drug delivery and biosensing applications [82], though their stability can decrease under high ionic strength or physiological conditions [86]. In contrast, chemically crosslinked hydrogels form permanent covalent networks that offer superior mechanical integrity and environmental stability [87]. Reactive amino groups facilitate crosslinking with agents such as genipin to produce biocompatible [88,89], slowly degradable gels [90,91], although with reduced reversibility and potential concerns about crosslinker toxicity or processing complexity [92]. Composite hydrogels have emerged as the dominant research focus. By integrating chitin or chitosan with other natural or synthetic polymers, or with inorganic phases, these composites combine biocompatibility with increased toughness, elasticity, or multifunctionality [21,93,94]. Natural polymer composites emphasise sustainability and biological performance, while synthetic and inorganic composites are designed to improve mechanical strength, durability, and functionalities such as conductivity or bioactivity [95,96]. In general, the wide diversity of chitin- and chitosan-based hydrogels allows for precise tailoring of the structure–property relationships, allowing the selection of the most appropriate hydrogel type based on the desired balance of reversibility, mechanical performance, environmental stability, and specific application requirements [97,98,99] (Table 1).

### 2.6. Structure–Property Relationships in Chitin/Chitosan Hydrogels

The macroscopic properties of chitosan hydrogels, such as gelation kinetics, mechanical strength, swelling behaviour, degradation rate, pore structure, and drug release, are strongly dictated by DDA, MW, crystallinity, source purity, and polymorphic form. DDA (typically 70–95% in hydrogel grade materials) primarily controls the density of protonatable glucosamine units. Higher DDA increases the cationic charge density below pKa (~6.5), enhancing ionic crosslinking with anionic agents, elevating storage modulus (G′) and producing more compact networks, although it can decrease equilibrium swelling in certain systems [100,101]. Moderate DDA values (75–85%) frequently optimise thermosensitive gelation (e.g., with β-glycerophosphate) by balancing hydrophobic interactions of residual acetyl groups with electrostatic repulsion, resulting in homogeneous gels with tunable sol–gel transition temperatures [52]. MW exerts a major influence through chain entanglement and solution viscosity. High-MW CS (>500 kDa) generates mechanically robust hydrogels characterised by smaller pore sizes, slower enzymatic degradation, and more sustained release profiles due to increased entanglement density [48,102].

On the contrary, low-MW CS enables faster gelation, higher swelling ratios, and accelerated release kinetics but often compromises long-term mechanical integrity. The synergistic effects between DDA and MW are particularly important; combinations such as high-MW with ~75% DDA produce compact, regular microstructures ideal for controlled drug delivery [52]. Crystallinity, which arises from intramolecular and intermolecular hydrogen bonding between hydroxyl and amino groups, further modulates hydration and mechanical performance. Higher crystallinity confers rigidity and thermal stability but restricts segmental mobility, thereby reducing swelling capacity and drug diffusion rates. Controlled processing methods, including thermomechanical plasticization or freeze-induced dehydration, can reduce overall crystallinity or trigger polymorphic transitions (e.g., form I hydrated form I to anhydrous Form II), improving flexibility and promoting self-association within microgel networks [103,104]. The purity and biological origin introduce additional critical variability. Crustacean-derived CS frequently contains residual proteins, minerals, or pigments that can interfere with gelation kinetics and biocompatibility, whereas fungal sources typically offer higher purity, lower allergenicity, and more consistent acetylation patterns, leading to better reproduce. Impurities can also skew the measured DDA and MW values, affecting batch consistency [59].

Finally, the polymorphic state influences the molecular packing efficiency. Transitions between crystalline forms, easily induced by pH adjustment, temperature, or freezing, directly alter the pore architecture and stimuli-responsive behaviour [103]. Overall, these interdependent parameters enable rational hydrogel design: high-DDA/high-MW/low-crystallinity CS is preferred for strong, slowly degrading tissue engineering scaffolds, while tailored lower-MW variants suit injectable or fast-swelling delivery systems [21,105]. Comprehensive characterisation and standardized reporting of these variables are essential for translational success.

**Table 1:** Classification of chitin- and chitosan-based hydrogels.

Hydrogel Type	Building Blocks	Crosslinking Mechanism	Characteristics	Advantages	Limitations	Applications	Ref.
Physical Ch hydrogels	Chitin	Hydrogen bonding, chain entanglement, hydrophobic interactions	Thermoreversible, anisotropic microstructures, reduced crystallinity	No toxic crosslinkers	Low mechanical strength, solvent sensitivity	Temporary scaffolds, model soft materials	[106,107]
ChNCs hydrogel	ChNCs	Physical percolation and electrostatic interactions	High transparency, rigid network, self-supporting	High stiffness at low solid content, optical clarity	Brittle at high loading, limited extensibility	Reinforcement networks, sensing materials	[78,108]
Chitosan hydrogels	Chitosan (acid-soluble)	pH-induced association, ionic interactions	pH-responsive, reversible gelation	Mild conditions, tunable properties	Weak stability	Drug delivery, wound dressings	[109]
Ionically crosslinked CS hydrogels	Chitosan/anions	Reversible electrostatic crosslinks	Improved strength, pH-sensitive swelling	Adjustable crosslink density, biocompatible	Ionic bonds	Controlled release, biosensors	[110]
Chemically crosslinked	Chitin	Covalent bonds	Permanent networks, low solubility	High stability, chemical resistance	Limited flexibility, harsh synthesis conditions	Separation of membranes, structural gels	[111,112]
Chemically crosslinked CS hydrogels	Chitosan	Covalent amide, Schiff base, ether bonds	Stable networks, controllable porosity/swelling	Strong, durable, tunable degradation	Potential cytotoxicity of some crosslinkers	Tissue engineering, implants	[113,114]
Ch/CS/composite hydrogels	Chitin/chitosan	Ionic, physical, or covalent interactions	Synergistic properties, improved toughness	Biobased, tunable mechanics	Batch variability	Biomedical and food-related uses	[115]
Ch/CS/synthetic polymer composite	Chitin/chitosan/PEG/PVA,	Grafting or interpenetrating networks	Enhanced elasticity and processability	Improved mechanical performance	Reduced biodegradability	Soft robotics, load-bearing gels	[116]
Ch/CS/inorganic composite hydrogels	Chitin/chitosan/inorganic phases	Physical embedding or in situ formation	High strength/Multifunctionality	Added conductivity and bioactivity	Increased brittleness or opacity	Bone repair, catalysis, sensors	[117,118]

Ch = Chitin; ChNCs = Chitin nanocrystals; CS = Chitosan.

### 2.7. Structural and Functional Engineering Strategies

To overcome intrinsic limitations of chitin- and chitosan-based hydrogels—such as low compressive strength (typically < 50 kPa for pristine hydrogels), rapid or uncontrolled enzymatic degradation, poor cell adhesion on unmodified surfaces, and limited osteoinductive bioactivity—researchers employ multifaceted structural and functional engineering strategies. Each modification is rationally designed to yield quantifiable improvements in key performance metrics [119] (Table 2). Hybrid and composite chitin/chitosan hydrogels represent a foundational approach. Combining chitin or chitosan with inorganic fillers such as nano-hydroxyapatite (n-HA), bioactive glass, or silica nanoparticles markedly reinforces the network. For example, incorporation of n-HA or graphene oxide-reinforced n-HA can raise compressive strength from ~0.3 MPa to 1.7–53 MPa (depending on load and additional reinforcements), while reducing degradation rates (for example, from 13% to 6% mass loss in 21 days) through densification and barrier effects [120,121].

These composites simultaneously boost osteoconductivity and mineral deposition, making them suitable for load-bearing bone applications. The mixing with natural polymers such as collagen, gelatin or alginate improves the mimicry of ECM, enhances hydrophilicity, and allows tunable degradation kinetics (often accelerating or stabilising resorption to match tissue regeneration of weeks to months), while increasing cell viability and adhesion through better integrin-binding motifs and porosity control [122,123] (Table 2). Biofunctionalization via covalent grafting or affinity-based immobilisation of bioactive motifs further optimises cellular responses. Grafting RGD peptides (Arg-Gly-Asp) onto chitosan backbones significantly promotes integrin-mediated cell adhesion, leading to enhanced spread, focal adhesion formation, and proliferation. Studies report 2- to 3-fold increases in attached cell density, higher expression of Ki-67 (indicating active proliferation), and improved migration rates (e.g., significantly higher wound closure in scratch assays, often >70% at 24 h with synergistic modifications) [124,125]. Controlled release or immobilisation of growth factors such as BMP-2, VEGF, or TGF-β enhances bioactivity by stimulating osteogenic differentiation, angiogenesis, and tissue-specific extracellular matrix production, with measurable outcomes that include increased alkaline phosphatase activity, calcium nodule formation, and vascular ingrowth [119].

Architectural engineering focusses on hierarchical pore structures. Techniques including freeze-drying, cryogelation, electrospinning, 3D bioprinting, and microfluidic methods allow precise control over pore size (typically 50–400 µm), interconnectivity, and porosity (>80–90%). Larger and interconnected pores (e.g., >90–200 µm) facilitate deeper cell infiltration (up to 400 µm or more), superior nutrient/oxygen diffusion, and vascularization, resulting in improved cell viability (~80% in deep layers) and proliferation over 14+ days [126,127]. Optimised architectures also balance mechanical integrity with swelling capacity, preventing collapse while supporting tissue growth.

Stimuli-responsive modifications introduce dynamic, adaptive behaviour. Incorporation of pH, temperature, enzyme, or light-sensitive linkages enables in situ gelation under physiological conditions, rapid self-healing (recovery of mechanical properties within minutes after shear damage), injectability, and on-demand release of therapeutics. These features produce controlled degradation profiles (e.g., enzyme-triggered rapid resorption in days when desired), sustained yet stimuli-triggered bioactive molecule delivery, and improved integration with irregular defect sites, while maintaining structural stability during the healing phase [128,129]. Collectively, these strategies transform chitin- and chitosan-based hydrogels into versatile, high-performance platforms. By directly linking modifications to measurable results such as compressive modulus/strength, degradation half-life, cell adhesion density and migration speed, proliferation rates, and bioactivity markers (e.g., ALP activity, mineralization), researchers can systematically optimise hydrogels for specific applications ranging from soft tissue regeneration and wound healing to load-bearing bone repair [20] (Table 2).

**Table 2:** Chitin/chitosan-based engineering strategies.

Material System	Strategy	Measurable Outcomes	Benefits	Limitations	Ref.
**Chitin**	Crosslinking & porous scaffold fabrication (freeze-drying, cryogelation)	High compressive strength, controlled pore size, high swelling ratio	Structural stability; biodegradability	Poor solubility; limited cell adhesion	[13,15,130]
**Chitin**	Composite with inorganic fillers (hydroxyapatite, silica)	High Young’s modulus, High mineralization, High osteoconductivity	Bone tissue engineering suitability	Brittleness; dispersion challenges	[122,130,131]
**Chitosan**	Ionic/covalent crosslinking (genipin, TPP)	Tunable degradation rate, High mechanical strength, controlled gelation	Injectable systems; antimicrobial activity	Weak mechanical strength alone	[8,132,133]
**Chitosan**	Blending with natural polymers (collagen, gelatin, alginate)	High cell adhesion, ↑ proliferation, improved cytocompatibility	ECM mimicry; enhanced bioactivity	Reduced stiffness; faster degradation	[8,132,134]
**Chitosan Derivatives** (carboxymethyl, quaternized)	Chemical modification for solubility & functionality	High solubility at physiological pH, High cell viability, and tunable degradation	Improved processing & biofunctionality	Complex synthesis; variability in substitution	[134,135]
**Chitosan Derivatives**	Biofunctionalization (RGD peptides, BMP-2, VEGF, TGF-β)	High cell adhesion, High differentiation markers, controlled release kinetics	Targeted tissue regeneration	Cost; biomolecule instability	[135,136]
**Nanocomposites (Chitin/Chitosan)**	Nanoparticle incorporation (hydroxyapatite, silica, bioactive glass, metal NPs)	High compressive strength, High stiffness, High bioactivity, antimicrobial effect	Synergistic mechanical–biological performance	Cytotoxicity risk; aggregation	[137]
**Nanocomposites**	Stimuli-responsive systems (pH, temperature, enzyme-sensitive)	Controlled degradation, self-healing, tunable drug release	Smart adaptive behaviour	Design complexity; reproducibility issues	[134,138]

### 2.8. Classification of Hydrogels Based on Chitin and Chitosan

**Figure 1:**
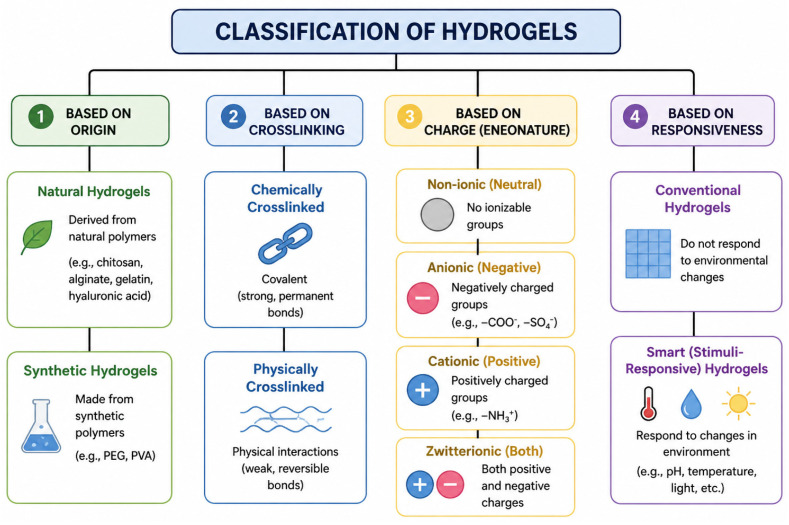
Classifications of chitin/chitosan hydrogels.

Hydrogels based on biopolymers chitin and chitosan are highly versatile polymeric networks that can be categorized based on multiple criteria that reflect their composition, structure, and functional properties [139]. This figure summarizes four principal classification schemes (Figure 1): (i) origin, distinguishing natural hydrogels derived from biopolymers such as chitosan, alginate, collagen, gelatin, and hyaluronic acid, from synthetic hydrogels synthesized from polymers like PEG, PVA, PAAm, and PNIPAM [140,141]; (ii) nature of crosslinking, which includes chemically covalent crosslinked hydrogels and physically crosslinked hydrogels stabilized by noncovalent interactions and cationic hydrogels with charged functionalities [141,142]; (iii) electrolytic properties, encompassing non-ionic, anionic, cationic, and zwitterionic gels that respond differently to pH and ionic strength [143]; and (iv) response to stimuli, separating conventional hydrogels with static behaviour from smart, stimuli-responsive hydrogels that undergo reversible changes in response to environmental triggers such as temperature, pH, or ionic strength [144,145].

### 2.9. Applications of Chitin/Chitosan-Based Hydrogels in Tissue Engineering

**Figure 2:**
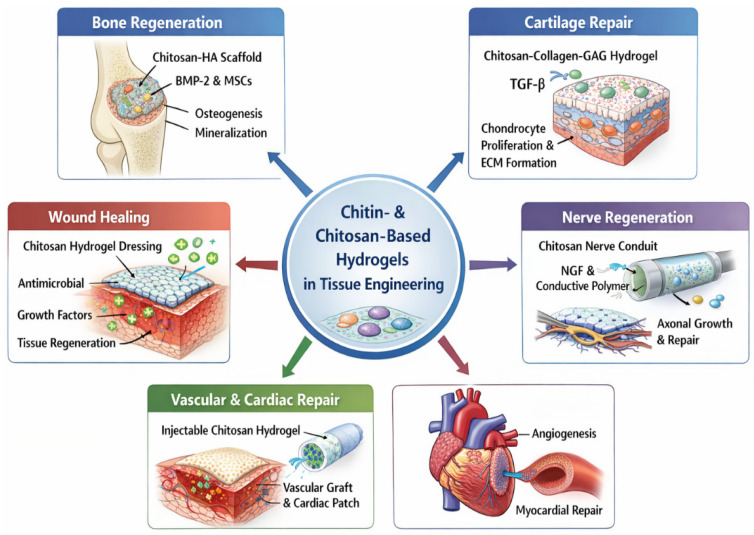
Applications of chitin/chitosan-based hydrogels in tissue engineering.

Chitin- and chitosan-based hydrogels have emerged as versatile scaffolds in tissue engineering due to their biocompatibility, biodegradability, antimicrobial activity, and structural likeness to extracellular matrix (ECM) components [119,146]. Their applications span bone, cartilage, skin, nerve, vascular, and soft tissue regeneration, often enhanced by chemical modification, composite formulation, and incorporation of bioactive molecules [147,148] (Figure 2).

For example, in bone tissue engineering (Table 3), it requires high compressive strength and osteoconductivity (typically in the MPa range), which requires the incorporation of inorganic fillers such as hydroxyapatite (HAp) or bioactive glass to improve stiffness and promote mineralization. In contrast, cartilage regeneration requires moderate mechanical strength combined with high elasticity and resistance to cyclic loading, where chitosan-based hydrogels are often optimised through polymer blending and controlled crosslinking to achieve appropriate viscoelastic properties. For soft tissues, such as skin and wound healing, the primary requirements shift toward high porosity, rapid degradation, and strong cell adhesion (Table 3). In these cases, chitosan hydrogels blended with collagen or gelatin demonstrate superior performance by enhancing fibroblast attachment and accelerating tissue regeneration, although they typically exhibit lower mechanical strength compared to bone-targeted systems. Similarly, vascular and cardiac tissues require highly interconnected porous structures and pro-angiogenic bioactivity, where biofunctionalization with growth factors such as VEGF becomes critical to promote vascularization (Table 3).

A key limitation across these applications is the trade-off between mechanical strength and biological performance. Systems optimised for bone regeneration often exhibit reduced degradation rates and limited flexibility, whereas hydrogels designed for soft tissues may lack sufficient mechanical stability. Consequently, the selection of chitin/chitosan-based systems must be tailored to tissue-specific requirements, balancing compressive strength, degradation kinetics, and bioactivity to achieve optimal regenerative outcomes.

#### 2.9.1. Chitin/Chitosan Hydrogels in Bone Tissue Engineering

Chitosan-based hydrogels are widely utilized in bone regeneration due to their capacity to incorporate osteoconductive inorganic fillers such as hydroxyapatite (HAp), tricalcium phosphate (TCP), and bioactive glass [153,154,155]. Inclusion of these bioactive ceramics improves the mechanical stiffness of the hydrogel matrix while promoting osteoblast adhesion, proliferation, and mineralization of the extracellular matrix [154,156]. In particular, chitosan–hydroxyapatite composite scaffolds have shown the ability to induce differentiation of mesenchymal stem cells (MSCs) into the osteogenic lineage while maintaining a controlled degradation rate that aligns with new bone formation [157,158] (Table 4). Furthermore, the incorporation of osteoinductive growth factors such as bone morphogenetic protein 2 (BMP-2) allows localized stimulation of bone formation, thus improving both the rate and quality of tissue regeneration [159,160].

**Figure 3:**
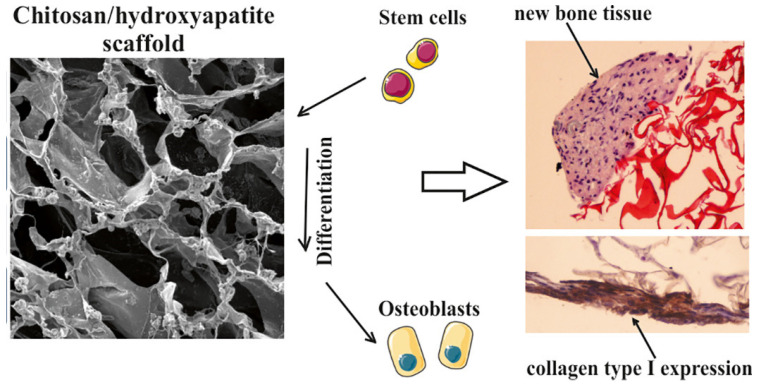
Osteogenic differentiation of human mesenchymal stem cells on a substituted calcium phosphate/chitosan composite scaffold. Reproduced from [173] with permission.

Beyond ceramic reinforcement, ionic substitution has emerged as a promising strategy to further improve the biological properties of calcium phosphates (CaP) and CaP-based composite scaffolds [170]. Although the influence of individual ionic dopants has been widely investigated, comprehensive studies that examine multisubstituted organic-inorganic systems remain relatively scarce [170,171]. Highly porous chitosan-based composite scaffolds incorporating calcium phosphates substituted with ions Sr^2+,^ Mg^2+,^ Zn^2+^, and SeO_3_^2−^ were fabricated using a freeze-gelation technique [172]. Structural analysis revealed a well-interconnected porous architecture with homogeneous dispersion of the substituted CaPs throughout the chitosan matrix. Importantly, the scaffolds maintained structural integrity during a 28-day degradation period, indicating favourable stability for bone tissue regeneration uses [172]. The osteogenic potential of hMSCs) cultured on these scaffolds was evaluated under both static and dynamic conditions. Histological evaluation, immunohistochemical staining and RT-qPCR analysis demonstrated that multiionic substitution significantly improved osteogenic differentiation [173] (Figure 3). Specifically, scaffolds containing multisubstituted CaPs exhibited increased expression of osteogenesis-related markers and greater phosphate deposition compared to scaffolds containing non-substituted CaPs [173]. Collectively, these findings highlight the synergistic potential of combining chitosan matrices with calcium phosphates to promote enhanced bone regeneration (Figure 3).

#### 2.9.2. Uses of Chitin/Chitosan Hydrogel in Cartilage Regeneration

Articular cartilage exhibits very limited self-repair capacity, which makes chitin- and chitosan-based hydrogels attractive scaffolds due to their ability to mimic key features of the native extracellular matrix (ECM). Beyond their high water content, which supports cell survival and matrix synthesis, their relevance for cartilage repair is primarily defined by their ability to replicate viscoelastic behaviour, lubricity, and long-term load-bearing performance, all of which are essential for functional joint restoration.

Viscoelasticity is a fundamental property of native cartilage, enabling it to dissipate energy under cyclic loading and resist mechanical fatigue. This behaviour is characterised by parameters such as storage modulus (G′), the loss modulus (G′), the relaxation of stress and creep response. Chitosan-based hydrogels inherently exhibit viscoelastic characteristics as a result of their hydrated polymer network, and these properties can be further tuned through crosslinking density, polymer blending, or formation of interpenetrating networks. For instance, combining chitosan with glycosaminoglycans (GAGs) or collagen not only helps mesenchymal stem cell (MSC) chondrogenesis but also improves stress relaxation behaviour and ECM deposition, both of which are critical for maintaining cartilage function under dynamic loading. Functionalisation with growth factors such as TGF-β or IGF-1 further enhances cartilage-specific matrix synthesis, including type II collagen and proteoglycans, which contribute to the intrinsic viscoelasticity [174,175]. In addition to bulk mechanical behaviour, lubricity is essential to minimise friction at articulating surfaces and prevent long-term degeneration. Native cartilage achieves extremely low friction coefficients through a hydrated surface rich in proteoglycans and lubricating molecules. Chitosan hydrogels can approximate this behaviour through their high content and incorporation of ECM components, such as GAGs, which help form hydration layers that reduce the coefficient of friction and wear rate. Injectable thermosensitive chitosan hydrogels further support this function by allowing conformal filling of irregular defects, thus restoring a smooth articulating surface and reducing mechanical abrasion in situ [174,175].

Long-term load-bearing performance is equally critical, as cartilage is subjected to repetitive compressive and shear forces during daily activity. Pure chitosan hydrogels, although biocompatible, often lack sufficient mechanical strength and fatigue resistance. To address this limitation, reinforcement strategies such as polymer blend, nanofiber incorporation, and composite design have been widely explored. For example, Zhao et al. developed chitosan hydrogels crosslinked with 1,6-diisocyanatohexane and polyethylene glycol, seeded with rabbit chondrocytes, which demonstrated complete defect repair, excellent integration, and cartilage-like histology after 12 weeks, achieving significantly higher ICRS scores than controls [176].

**Figure 4:**
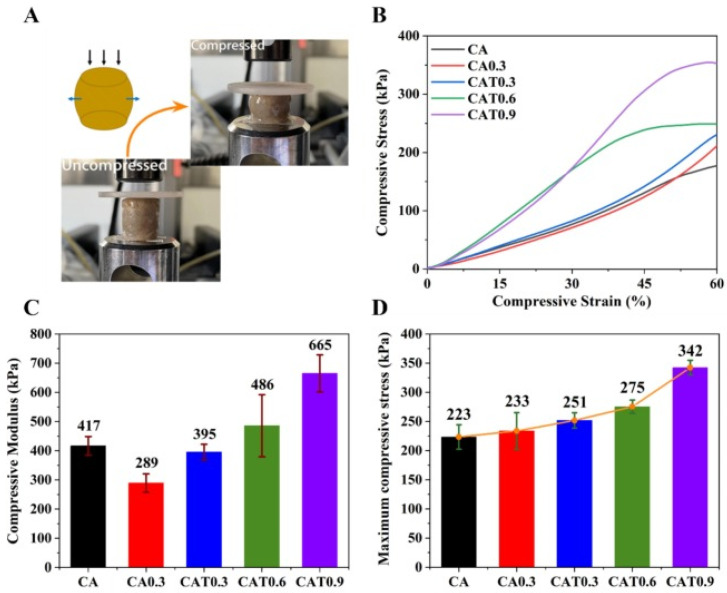
(**A**) State diagram before and after bracket compression. (**B**) The stress–strain curves. (**C**) The compressive modulus. (**D**) Maximum compressive stress of CA, CA0.3, CAT0.3, CAT0.6, and CAT0.9. Reproduced from [177] with permission.

These outcomes suggest not only improved biological performance but also sufficient mechanical stability under in vivo loading conditions. Further improvements in load-bearing capacity have been achieved through composite approaches. Chitosan combined with cartilage-derived ECM promotes stem cell differentiation, increasing COL_2_A_1_ and ACAN expression, along with abundant type II collagen and proteoglycan deposition, which are essential to resisting compressive forces and maintaining tissue integrity [176]. Similarly, Shen et al. reinforced citric acid-modified chitosan hydrogels with short electrospun PLGA fibres and decellularized cartilage matrix, significantly improving compressive modulus, structural stability, and fatigue resistance while supporting chondrocyte adhesion and proliferation, ultimately leading to effective osteochondral repair in a rabbit model [176] (Figure 4). In another approach, sodium alginate/chitosan hydrogels reinforced with biodegradable nanofibers (PLGA, PCL, gelatin) exhibited improved compressive stress and modulus, demonstrating that nanofiber integration can improve mechanical durability and recovery after cyclic loading while preserving biocompatibility [177] (Figure 4).

#### 2.9.3. Uses of Chitin/Chitosan Hydrogel in Wound Healing

**Figure 5:**
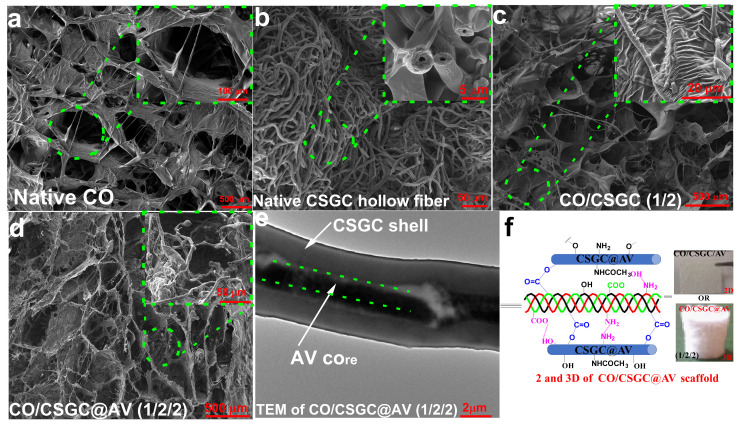
Functional wound dressing (FWD). (**a**) SEM of native collagen (CO); (**b**) SEM of native Chitosan-glucan complex hollow fibers (CSGC); (**c**) wound dressing of CO/CSGC; (**d**) Wound dressing of CO/CSGC/AV (1/2/2); (**e**) TEM of CO/CSGC/AV (1/2/2); (**f**) Interaction between CO/CSGC/AV. Reproduced from [180] with permission.

Chitosan’s antimicrobial and hemostatic properties make it ideal for wound dressings. Chitosan hydrogels accelerate wound closure, reduce bacterial colonisation, and stimulate tissue regeneration by supporting keratinocyte and fibroblast proliferation [178]. Hydrogels can also act as carriers of bioactive molecules, such as VEGF, to enhance angiogenesis or antibiotics for infection control. Composite chitosan–alginate or chitosan–gelatin hydrogels have been shown to improve mechanical stability and maintain a moist wound environment, which is critical for faster healing [179]. New composite wound scaffolds were fabricated via combining collagen (CO) with hollow fibres of the chitosan/glucose complex (CSGC) and incorporated aloe vera (AV) by freeze-drying. Inclusion of CSGC and AV improved physicochemical properties compared to collagen alone, supporting the potential for wound dressing and regeneration [30] (Figure 5). The same CO/CSGC@AV dressing was evaluated for biological performance. It showed excellent biocompatibility with human dermal fibroblasts, enhanced swelling/hydrolytic stability, significant antibacterial activity, and in vivo [180] (Figure 5).

In addition, the Amritha Vijayan group has developed chitosan scaffolds chemically crosslinked with polyethylene glycol (PEG) as delivery platforms for bioactive molecules in wound healing. By incorporating growth factors such as basic fibroblast growth factor (bFGF) and vascular endothelial growth factor (VEGF) into the chitosan/polyethylene glycol (CS/PEG) matrix, the scaffolds provided controlled and sustained release at the wound site. In vitro and in vitro studies showed that these functionalized scaffolds promoted fibroblast and endothelial cell migration, stimulated angiogenesis, and supported extracellular matrix deposition. The combined effect accelerated wound closure, improved tissue granulation, and improved collagen remodelling, effectively advancing the healing process from the proliferative phase to the remodelling phase. Such multifunctional CSPEG growth factor scaffolds demonstrate the potential of biopolymer-based composite systems for controlled therapeutic delivery in skin tissue regeneration [181].

The antibacterial activity of chitin and chitosan is primarily attributed to their cationic amino groups, which become protonated under physiological conditions [30,33,37,43]. These positively charged groups interact electrostatically with negatively charged bacterial membranes, leading to membrane disruption, increased permeability, and leakage of intracellular components, resulting in bacterial cell death or growth inhibition. In addition, low-molecular-weight chitosan can penetrate microbial cells and bind to DNA, inhibiting transcription and replication processes [182]. The efficacy is strongly influenced by the degree of deacetylation, molecular weight, and pH-dependent solubility, which determine charge density and interaction strength [183]. Chitin contributes indirectly through its immunomodulatory activity, enhancing innate immune responses that reduce microbial colonisation at wound sites [184]. The hemostatic activity of chitin and chitosan results from electrostatic interactions with blood components [10,180]. Chitosan rapidly binds to negatively charged red blood cells and platelets, promoting cell aggregation and platelet activation, which accelerates clot formation independent of the intrinsic coagulation cascade. This makes it effective even under coagulopathic conditions [180]. Measurable outcomes include reduced clotting time, increased clot strength, and faster bleeding cessation. Additionally, the porous hydrogel structure improves fluid absorption and local concentration of clotting factors, further supporting rapid haemostasis [185]. Chitin also contributes by promoting platelet adhesion and activation at wound interfaces, enhancing initial stabilisation of the clot stabilization [185].

Chitin and chitosan also actively promote angiogenesis [184], a critical process in tissue regeneration. These biomaterials modulate macrophage polarisation toward a pro-healing phenotype, leading to increased secretion of pro-angiogenic cytokines such as VEGF and FGF [15,69]. This cascade enhances endothelial cell migration, proliferation, and capillary formation, which can be quantified by increased vascular density and CD31 expression. Furthermore, chitin degradation products (e.g., N-acetylglucosamine oligomers) can directly stimulate fibroblasts and endothelial cells, further improving angiogenic signalling [4,9]. Chitosan-based systems can also be engineered for the controlled release of growth factors or bioactive ions, extending angiogenic stimulation over time [15]. These mechanisms act in a coordinated sequence during wound healing: antimicrobial activity reduces infection risk, hemostatic action stabilises the wound environment, and angiogenesis supports tissue regeneration and remodelling. Synergy among these processes significantly accelerates wound closure and improves tissue quality [15].

#### 2.9.4. Applications of the Chitin/Chitosan Hydrogel in Nerve Tissue Engineering

Peripheral nerve regeneration requires scaffolds that recreate the highly organised and electrically active microenvironment of native nerve tissue. Chitin- and chitosan-based hydrogels are widely used because of their biocompatibility and the ability to be engineered for axonal guidance, Schwann cell support, and electrical functionality. Aligned structural cues are essential for directing neurite extension and preventing random axonal sprouting. Chitosan-based conduits can be manufactured with aligned fibres, microchannels, or gradient porous architectures, which enhance directed neurite outgrowth and functional regeneration [186,187]. Such anisotropic structures significantly improve axon elongation, regeneration density, and nerve conduction velocity by providing physical contact guidance similar to native endoneurial tubes [188]. Native nerve tissue is dependent on electrochemical signaling; therefore, conductivity is a critical design requirement. Pure chitosan is insulating, but integration with conductive materials such as graphene oxide, carbon nanotubes, and PEDOT introduces electrically active pathways that improve neuronal depolarization, synaptic signaling, and neurite extension [189,190]. Conductive scaffolds also improve responsiveness to external electrical stimulation, further accelerating axonal growth and maturation [6,191]. Schwann cells play a central role in peripheral nerve regeneration through axonal guidance, myelination, and secretion of neurotrophic factors. Chitosan-based matrices promote the adhesion and proliferation due to their ECM-like properties and positive charge [147].

**Figure 6:**
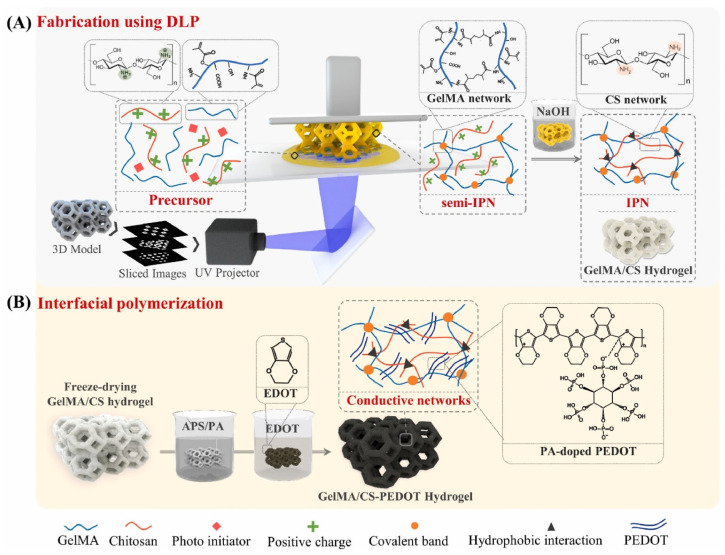
Schematic of synthesis of the conducting GelMA/CS-PEDOT hydrogel. (**A**) DLP printing of GelMA/CS ink into the 3D structure, followed by a two-step crosslinking strategy to obtain the GelMA/CS IPN hydrogel. (**B**) Interfacial polymerization process in forming PA-doped conductive PEDOT hydrogel. Reproduced from [196] with permission.

Incorporation of collagen or neurotrophic factors further enhances Schwann cell migration and myelin formation, which is generally assessed by MBP expression and thickness of the myelin sheath thickness [192]. In a representative study, Schwann cell-loaded collagen nerve conduits significantly improved motor recovery, axonal regeneration, and myelination in a rat sciatic nerve model compared to acellular and silicone controls, highlighting the critical role of cellular incorporation [193]. Neurotrophic factors such as NGF and BDNF improve neuronal survival and axonal extension by activating Trk receptor-mediated signaling pathways, thus increasing neurite length, axonal density, and synaptic maturation [194]. Chitosan hydrogels enable sustained delivery of these factors, improving long-term regenerative outcomes [195]. Hybrid conductive systems such as chitosan–graphene oxide and GelMA/chitosan–PEDOT scaffolds provide both physical guidance and electrical stimulation capabilities, enhancing neural network formation and functional regeneration [196] (Figure 6). In vivo studies demonstrate improved regeneration in critical nerve gaps, confirming their translational potential [197].

#### 2.9.5. Chitin and Chitosan Hydrogel for Vascular and Cardiac Tissue Engineering

Chitin- and chitosan-based hydrogels have emerged as promising platforms for the engineering of vascular and cardiac tissue due to their biocompatibility, tunable structure, and capacity for functional modification. However, successful application in these tissues requires careful consideration of four critical design requirements: hemocompatibility, endothelialization, electrical conductivity, and mechanical pulsatile mechanical performance [198]. Hemocompatibility is essential for vascular applications, as implanted materials are in direct contact with blood. Chitosan exhibits generally favourable blood compatibility due to its hydrophilic nature and ability to form hydrated surfaces that reduce protein adsorption and thrombogenicity. However, its intrinsic positive charge can also promote platelet adhesion; therefore, surface modification or mixing with other polymers (e.g., heparin, gelatin) is often used to improve anticoagulant behaviour and reduce platelet activation, as evaluated by platelet adhesion assays and coagulation time measurements [198,199].

Equally important is endothelialization, which ensures the formation of a functional endothelial layer to maintain vascular homeostasis and prevent thrombosis. Chitosan hydrogels provide an ECM-like microenvironment that supports endothelial cell adhesion, proliferation, and migration. Incorporation of angiogenic growth factors such as VEGF and FGF further enhances endothelialization and neovascularisation, typically measured by increased endothelial cell density, nitric oxide production, and CD31 expression [15,200]. Tubular chitosan-based scaffolds and injectable hydrogels have shown potential for small-diameter vessel regeneration, where rapid endothelial coverage is critical to long-term patency of the graft. In cardiac tissue engineering, electrical conductivity is a key requirement because the native myocardium relies on synchronised electrical signalling for coordinated contraction. Conventional polymeric scaffolds are electrically insulating, which can disrupt signal propagation and increase the risk of arrhythmias.

To overcome this limitation, chitosan-based hydrogels have been functionalised with conductive nanomaterials such as carbon nanotubes, graphene derivatives, or conductive polymers (e.g., polypyrrole, PEDOT). These additives create conductive networks within the hydrogel, enhancing electrical coupling between cardiomyocytes, propagation of action potentials, and contraction synchrony, as demonstrated by improved conduction velocity and expression of gap junction proteins such as connexin-43 [190,201]. For example, the incorporation of carbon nanotubes into chitosan/gelatin matrices has been shown to significantly improve electrical properties and support cardiomyocyte function, with conduction behaviour approaching that of native myocardium [201]. Another critical aspect is the pulsatile mechanical performance, since both vascular and cardiac tissues are subjected to dynamic mechanical loading. Hydrogels must exhibit appropriate elasticity, compliance, and fatigue resistance to withstand cyclic strain without mechanical failure. Chitosan-based systems can be engineered through crosslinking, composite formation, or fibre reinforcement to achieve mechanical properties compatible with native tissues, typically evaluated by elastic modulus, strain recovery, and cyclic loading tests. However, increasing stiffness to improve mechanical strength can compromise cell viability and electrical performance, highlighting the need for a balanced design [201].

#### 2.9.6. Other Emerging Applications

Beyond their established roles in bone, cartilage, skin, and nerve regeneration, chitin- and chitosan-based hydrogels are increasingly being explored in emerging and cross-disciplinary biomedical applications, owing to their tunable physicochemical properties, biocompatibility, and ease of functionalisation. One prominent area is advanced drug delivery, where these hydrogels serve as localised depots capable of controlled and stimuli-responsive release of therapeutic agents. Their adjustable swelling behaviour, network porosity, and degradability enable precise regulation of drug diffusion kinetics and release profiles, supporting applications ranging from sustained chemotherapy delivery to protein and gene therapeutics [202,203]. In addition, chitosan hydrogels are gaining traction in biosensing and bioelectronic interfaces. Their ability to incorporate conductive components and biological recognition elements allows the development of flexible, biocompatible sensing platforms to monitor physiological signals or detect biomarkers in real time [204]. These systems benefit from the inherent compatibility of chitosan with biological environments, enabling stable integration with living tissues.

Another emerging direction involves soft tissue and organoid engineering, where chitosan-based hydrogels provide a hydrated, ECM-mimicking microenvironment that supports diverse cell types. Their ability to encapsulate cells and bioactive molecules facilitates cell proliferation, differentiation, and tissue-like organisation, making them suitable for in vitro tissue models and regenerative applications [205]. Collectively, the versatility of chitin and chitosan hydrogels in these emerging fields is largely attributed to their tailorable swelling behaviour, porosity, and response to stimuli, which enable dynamic interactions with cells and biomolecules. However, further optimisation of mechanical stability, long-term functionality, and scalable fabrication remains necessary to fully translate these systems into clinical and industrial applications.

### 2.10. Grafting vs. Polyelectrolyte Complexation in Chitin/Chitosan Hydrogels

Chitosan has two principal reactive sites that allow chemical modification: the primary amino groups located in the deacetylated glucosamine units and the hydroxyl groups at the C3 and C6 positions of the acetylated and deacetylated residues. Covalent functionalisation at these sites yields grafted chitosan derivatives, which are well evidenced by spectroscopic techniques such as Fourier transform infrared (FTIR) and nuclear magnetic resonance (NMR). Grafting significantly broadens the physicochemical and biological performance of chitosan, improving properties such as metal ion chelation, aqueous and organic solubility, antimicrobial activity, and adsorption capacity, while maintaining its intrinsic mucoadhesivity, biocompatibility, and biodegradability [206,207,208].

**Figure 7:**
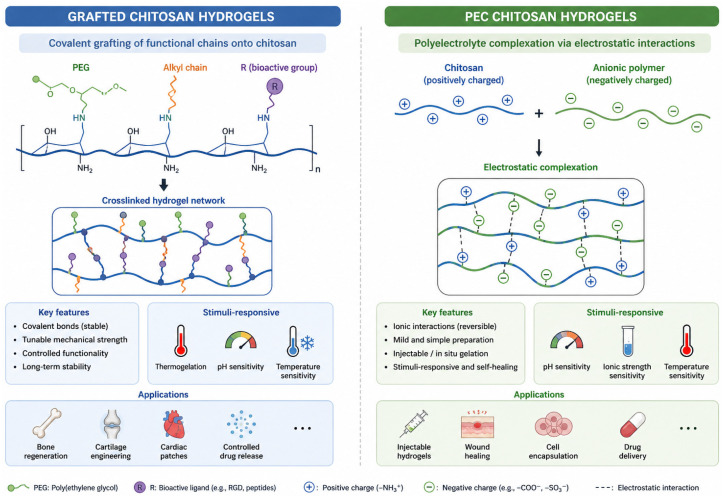
Two major strategies for functionalizing chitosan hydrogels: Grafting and polyelectrolyte complexation process.

Hydrogel formation via grafting generally proceeds through a two-stage mechanism: (i) covalent incorporation of functional moieties onto the polymer backbone, followed by (ii) assembly of the supramolecular network driven by secondary interactions among the grafted chains and/or native chitosan segments [21,209]. These secondary forces, such as hydrogen bonding and hydrophobic interactions, provide additional physical stabilisation of the hydrogel network (Figure 7). For example, poly(ethylene glycol) (PEG) grafts stabilize networks via hydrogen bonding, while alkyl chains, aldehyde-derived linkers, 2-hydroxyethyl methacrylate, polyacrylic acid, and quinone-based grafts promote hydrophobically driven associations [181,210]. Certain grafts further impart stimuli-responsive behaviour: Pluronic-modified chitosan exhibits thermogelling behaviour through temperature-induced chain entanglement, while poly(N-isopropylacrylamide) (PNIPAM) grafts introduce lower critical solution temperature (LCST) behaviour, leading to reversible sol–gel transitions around 32 °C. Similarly, carboxymethylation enhances intermolecular ordering and facilitates more efficient network formation [23,211].

In parallel, polyelectrolyte complex (PEC) hydrogels are formed through purely electrostatic interactions between cationic chitosan and anionic polymers, particularly glycosaminoglycans (GAGs) such as chondroitin sulphate and hyaluronic acid [212]. These physically crosslinked networks are capable of stabilising cells and enzymes while maintaining permeability for nutrients, metabolites, and bioactive molecules (e.g., dexamethasone and L-ascorbic acid). PEC hydrogels exhibit tunable swelling, porosity, and mechanical properties, and their stability can be further enhanced via secondary ionic crosslinking, avoiding the use of potentially cytotoxic covalent crosslinkers and therefore improving biocompatibility [213]. Structurally, chitosan–GAG PECs closely mimic the native extracellular matrix of cartilage, promoting chondrocyte adhesion, proliferation, and deposition of extracellular matrix while partially protecting GAG components from enzymatic degradation. In vitro studies have shown their utility as scaffolds for autologous chondrocyte culture and cartilage-like tissue engineering [214]. Similarly, chitosan–GAG systems support keratinocyte proliferation and accelerate wound closure in vivo without inducing significant inflammatory responses. When combined with collagen or additional GAG components, these systems further enable coculture strategies involving keratinocytes and fibroblasts for skin tissue reconstruction (Figure 7).

In particular, in some in vivo contexts, chitosan alone has shown superior performance compared to PECs containing GAG, indicating that formulation optimisation is critical. PECs formed with non-GAG polyanions retain the inherent antimicrobial and wound-healing properties of chitosan, providing multifunctional hydrogel dressings that promote tissue regeneration, prevent infection, maintain a hydrated microenvironment and act as drug delivery reservoirs [215].

Overall, structural modification of chitin and chitosan hydrogels is primarily achieved through covalent grafting or polyelectrolyte complexation, each governed by fundamentally different interaction mechanisms and resulting in distinct material behaviours. Grafting introduces permanent covalent modifications to amino or hydroxyl groups, enabling precise control over network architecture, crosslinking density, and surface chemistry. This allows for customised adjustment of mechanical strength, degradation kinetics, hydrophilicity, and bioactivity. For example, PEG grafting enhances elasticity and reduces immunogenicity, while peptide conjugation (e.g., RGD sequences) promotes cell adhesion through integrin-mediated pathways. Because these modifications are irreversible under physiological conditions, grafted systems offer long-term structural stability, making them suitable for load-bearing scaffolds and sustained-release biomedical implants. However, grafting often requires chemical reagents and reaction conditions that may compromise cytocompatibility, and excessive functionalisation may attenuate the native bioactivity of chitosan.

In contrast, PEC hydrogels rely on reversible electrostatic interactions between oppositely charged polymers such as chitosan and alginate, hyaluronic acid, or DNA. Their formation is governed by parameters including pH, ionic strength, and charge density, which collectively determine network stability and responsiveness. The dynamic nature of ionic interactions enables stimuli-responsive behaviour, self-healing capacity, and reversible swelling, making PEC systems particularly suitable for injectable formulations and controlled drug delivery platforms. Moreover, PEC formation occurs under mild, aqueous conditions without chemical crosslinkers, thereby preserving the bioactivity of encapsulated cells and growth factors. However, these advantages come at the cost of reduced mechanical strength and limited stability under physiological ionic fluctuations. Consequently, the choice between grafting and PEC strategies depends on the required balance between structural stability and functional adaptability: grafting is preferred for durable, mechanically robust, and highly engineered biomaterials, while PEC systems are favoured for dynamic, cell-compatible, and minimally processed hydrogel applications (Table 5).

### 2.11. Advanced Functional Chitosan-Based Hydrogels

#### 2.11.1. Magnetic Chitosan Hydrogels

**Figure 8:**
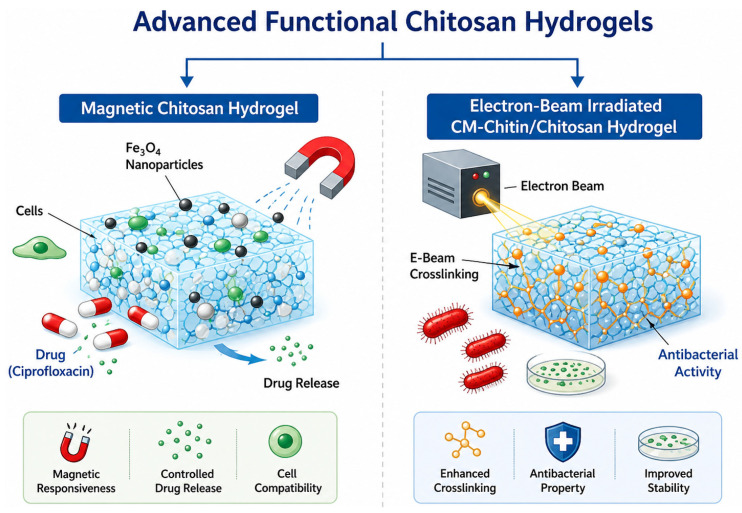
Advanced functional chitin/chitosan hydrogels.

Chitosan-based magnetic hydrogels with ordered architectures have emerged as promising platforms for cell culture and tissue engineering, offering physical guidance signals that mimic the native mechanical microenvironment [216,217]. Incorporation of magnetic structures allows directional control of cell growth and modulation of cellular behaviour, providing advantages over conventional hydrogels. Magnetic hydrogel scaffolds were fabricated from 1% chitosan, with 10% gelation and 1% hyaluronic acid as supporting polymers [216]. Glutaraldehyde was used as a chemical crosslinker, while Fe_3_O_4_ iron oxide nanoparticles imparted magnetic functionality (Figure 8). Scanning electron microscopy (SEM) revealed rough, irregular surfaces with interconnected three-dimensional porous networks, with pore size and porosity strongly dependent on polymer composition and crosslinking density. Mechanical testing demonstrated enhanced scaffold strength, with the breaking load increasing from 1.361 to 4.98 Kg as the glutaraldehyde concentration increased from 0.2 to 0.8 mL. These hydrogels also exhibited a pronounced antibacterial activity, achieving inhibition rates of 54–97% against *Staphylococcus aureus* and 26–92% against *Escherichia coli*. Drug delivery studies using ciprofloxacin hydrochloride showed sustained release over ~10 h, with iron oxide nanoparticles accelerating the release kinetics. These findings highlight the potential of magnetic chitosan-based hydrogels for multifunctional biomedical applications, which combine structural support, antibacterial activity, and controlled drug delivery [216].

#### 2.11.2. Electron-Beam-Irradiated Carboxymethyl Chitin and Chitosan Hydrogels

Carboxymethyl derivatives of chitin (CM-chitin) and chitosan (CM-chitosan) can be converted into hydrogels by irradiation of electron beams in aqueous media under vacuum [218,219]. In Long Zhao’s study, hydrogel formation occurred under paste-like conditions, whereas irradiation in dilute or solid states predominantly induced polymer degradation. Transparent hydrogels formed at doses <20 kGy, with the gel fraction strongly dependent on the polymer concentration. At low concentrations (<10 wt.%), chain scission dominated, while higher concentrations promoted crosslinking; optimal gel fractions for CM-chitin (60–70%) were achieved at 30–40 wt., and for CM-chitosan (50–60%) at 25–35% by weight. Excess concentrations (~50 wt.%) resulted in heterogeneous networks with reduced gel formation. Mechanical characterisation at 30 wt.% polymer showed that tensile strength increased with the irradiation dose, peaking at 0.45 MPa for CM-chitin (75 kGy) and 0.73 MPa for CM-chitosan (50 kGy), before declining due to network degradation. The elongation at break decreased with dose, reflecting increased brittleness. Both the CM-chitosan hydrogels and their soluble fractions showed antibacterial activity against *E. coli*, with only crosslinked networks contributing to bacterial suppression [218]. These results demonstrate that electron beam irradiation can be used to fine-tune hydrogel formation, mechanical properties, and bioactivity, highlighting its potential for diverse biomedical applications, such as tissue scaffolding and antimicrobial wound dressings.

#### 2.11.3. Injectable Hydrogels for Tissue Engineering

**Figure 9:**
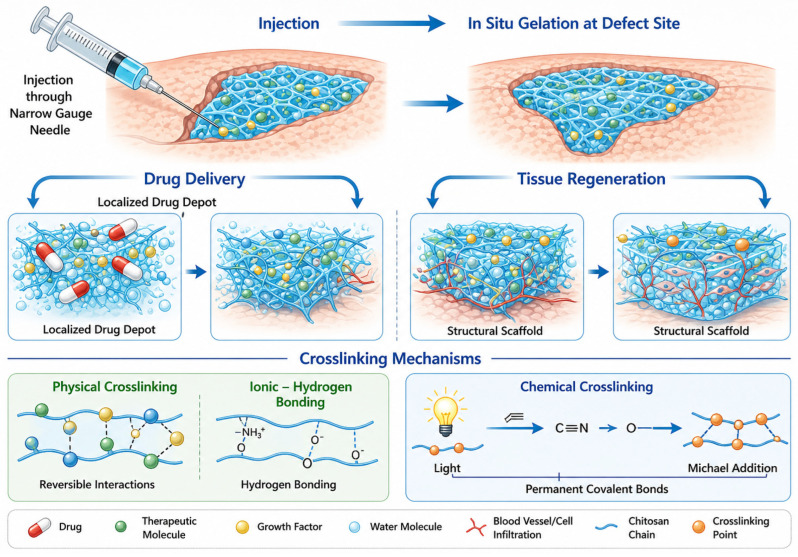
Injectable chitin/chitosan hydrogels for tissue engineering.

Injectable hydrogels have gained considerable attention as adaptable biomaterial systems due to their ability to be delivered in a minimally invasive manner and subsequently transformed into stable three-dimensional networks at the target site [220]. Their injectability arises from shear-responsive flow behaviour, allowing administration through narrow-gauge needles, followed by gelation in situ within a predefined time window [221,222]. This injectable to solid transition allows these materials to serve dual roles as localized drug delivery depots and structural scaffolds for supporting cartilage and bone tissue regeneration [223]. Furthermore, in situ formation hydrogels can conform to irregular defect geometries, offering a distinct advantage over pre-shaped implants [224]. The effectiveness of injectable hydrogels in regenerative applications is highly dependent on both the molecular composition of the polymer system and the approach used to construct the crosslinked network (Figure 9). To date, a wide range of polymeric materials have been explored, encompassing naturally derived macromolecules such as chitosan, collagen, gelatin, alginate, hyaluronic acid, heparin, and chondroitin sulphate, alongside synthetic polymers, including poly (ethylene glycol) (PEG) and poly (vinyl alcohol) (PVA).

These materials offer varying degrees of biocompatibility, degradability, and mechanical tunability, allowing customization for specific tissue engineering requirements [223,225,226]. From a fabrication perspective, injectable hydrogels can be formed via non-covalent or covalent interactions [227]. Physically assembled hydrogels rely on reversible forces such as ionic interactions, hydrogen bonding, or hydrophobic associations, resulting in dynamic and stimuli-responsive matrices [228]. In contrast, chemically crosslinked hydrogels are generated through permanent covalent bonds, which typically provide improved mechanical stability and structural integrity [229]. Based on the gelation mechanisms used, injectable hydrogel systems can be categorised into enzyme-triggered, light-activated, Schiff base–linked, Michael addition-mediated, click chemistry-based, and environmentally responsive hydrogels that react to changes in ionic strength, pH, or temperature changes [230]. Although injectable hydrogel technologies have been extensively investigated in recent decades, their clinical adoption in cartilage and bone regeneration remains limited [231]. Key challenges include achieving precise control over gelation kinetics, ensuring sufficient mechanical strength under physiological loading, maintaining long-term bioactivity, and matching degradation rates with tissue formation (Figure 9). Consequently, the rational design of injectable hydrogels that integrate injectability, biological performance, and mechanical functionality continues to be an urgent focus in research on musculoskeletal tissue engineering research [232].

## 3. Conclusions

Chitin and chitosan hydrogels have emerged as exceptionally versatile biomimetic scaffolds for tissue engineering owing to their inherent biocompatibility, biodegradability, structural similarity to glycosaminoglycans in the extracellular matrix, and intrinsic antimicrobial and hemostatic properties. The extensive chemical versatility of these polysaccharides, particularly the reactive amino and hydroxyl groups of chitosan, enables precise tailoring of the network architecture through physical, ionic, and covalent crosslinking strategies, as well as the formation of advanced composites, grafted derivatives, and polyelectrolyte complexes. Recent advances in nanostructural reinforcement (e.g., chitin nanowhiskers and nanocrystals), biofunctionalization with growth factors and peptides, and sophisticated fabrication routes (freeze drying, 3D bioprinting, electron beam irradiation, and in situ gelation) have produced scaffolds with controlled porosity, tunable mechanical properties, stimuli-responsive behaviour, and multifunctionality.

These platforms have shown substantial promise across diverse applications, including osteogenesis with ion-substituted calcium phosphates, chondrogenesis in cartilage-mimetic composites, accelerated wound closure with antimicrobial dressings, guided neural regeneration via conductive conduits, and electrically synchronised cardiac patches. However, several challenges remain, including limited mechanical robustness in load-bearing applications, batch-to-batch variability in material properties, and difficulties in scaling up and achieving regulatory approval for clinical translation. Collectively, chitin- and chitosan-based hydrogels represent sustainable bioderived platforms capable of recapitulating key aspects of native tissue microenvironments while supporting cell adhesion, proliferation, differentiation, and controlled bioactive-molecule delivery. Addressing current limitations through continued multidisciplinary research will be essential to fully realise their clinical potential.

## 4. Future Perspectives

Despite strong advances, the clinical translation of chitin- and chitosan-based hydrogels is still limited by a few key challenges. The most critical near-term issue is poor standardisation of material properties, including variability in molecular weight, degree of deacetylation, and impurity profiles, which leads to inconsistent performance across studies. Closely related is the need for robust long-term in vivo evidence, particularly with respect to degradation behaviour, immunological response, and functional tissue integration under physiological loading conditions. Therefore, in the short-term, research should prioritize standardized production protocols and mechanically reinforced hydrogel systems capable of maintaining stability in dynamic biological environments. Establishing reproducible material benchmarks will be essential for meaningful comparison and regulatory progression. In the mid-term, the focus should shift toward functional enhancement through hybrid bioengineering, including the incorporation of conductive, magnetic, or piezoelectric nanomaterials to enable externally controlled cell modulation and improved tissue regeneration. Parallel efforts should integrate these hydrogels with advanced biological platforms such as stem cells, organoids, and bioreactors to better replicate human physiological conditions. In the long-term, the field is expected to move toward intelligent, patient-specific regenerative systems, combining 4D bioprinting, multi-responsive hydrogels, and on-demand therapeutic delivery. Successful clinical translation will ultimately depend on aligning these innovations with standardised preclinical models and streamlined regulatory pathways.

## Figures and Tables

**Table 3:** Comparative Performance of Chitin/Chitosan Hydrogels across Tissue Types.

Tissue Type	Key Requirements	Chitin/Chitosan Strategy	Measurable Outcomes	Advantages	Limitations	Ref.
**Bone**	High compressive strength (MPa), osteoconductivity, slow degradation	Nanocomposites with hydroxyapatite, bioactive glass	High compressive strength, High Young’s modulus, high mineralization (ALP activity)	Strong mechanical support; promotes bone regeneration	Brittleness; slow degradation	[134,149]
**Cartilage**	Moderate strength, elasticity, resistance to cyclic loading	Crosslinked chitosan blends, viscoelastic tuning	High modulus, High resilience, improved ECM production	Good load distribution; mimics cartilage	Limited long-term durability	[150]
**Skin/Wound**	High porosity, fast degradation, strong cell adhesion	Chitosan/collagen/gelatin, porous scaffolds	High cell adhesion, High proliferation, faster degradation rate	Excellent healing; high biocompatibility	Low mechanical strength	[151]
**Cardiac/Vascular**	Elasticity, angiogenesis, bioactivity	Biofunctionalized hydrogels (e.g., VEGF delivery)	High angiogenesis, High cell viability, controlled release kinetics	Promotes vascular integration	Complex design; stability issues	[152]

**Table 4:** Nanocomposites chitosan-based hydrogels.

Composite System	Incorporated Component	Key Findings	Biological Outcome	Ref.
Chitosan/Hydroxyapatite (HA)	Osteoconductive calcium phosphate	Enhanced compressive strength and biomimetic mineral phase; supported osteogenic differentiation of MSC osteogenic differentiation	Increased ALP activity, osteocalcin expression, and mineral deposition	[161]
Chitosan/Tricalcium Phosphate (TCP)	Biodegradable CaP ceramic	Improved mechanical stability and controlled biodegradation	Promoted osteoblast proliferation and bone tissue formation	[162,163]
Chitosan/Bioactive Glass	Silicate-based bioactive filler	Ion release (Si, Ca, P) stimulated osteogenic signaling; improved scaffold stiffness	Enhanced cell proliferation and osteogenic marker expression	[164,165]
Chitosan/HA & BMP-2	Osteoconductive/osteoinductive system	Sustained BMP-2 release; synergistic scaffold–growth factor effect	Accelerated MSC differentiation and bone formation in-vivo	[166,167]
Chitosan/Growth Factors (e.g., BMP-2)	Osteoinductive biomolecule delivery	Controlled local release minimized systemic exposure	Increased osteogenic gene expression and improved bone repair quality	[168,169]

**Table 5:** Comparison between grafting and polyelectrolyte complexation on performance properties of hydrogels.

Strategy	Mechanism	Key Properties	Advantages	Limitations	Applications
**Grafting**	Covalent bonding to chitosan backbone	High stability, tunable mechanics, controlled bioactivity	Durable, precise functionalization	Complex chemistry, possible cytotoxicity	Bone, long-term implants
**Polyelectrolyte Complexation**	Electrostatic interactions between charged polymers	Dynamic, reversible, stimuli-responsive	Mild fabrication, injectable, self-healing	Lower strength, less stable in vivo	Drug delivery, soft tissue, injectable gels

## Data Availability

No new data were created or analyzed in this study. Data sharing is not applicable to this article.
